# Chronic Propafenone Application Increases Functional K_IR_2.1 Expression In Vitro

**DOI:** 10.3390/ph16030404

**Published:** 2023-03-07

**Authors:** Encan Li, Willy Kool, Liset Woolschot, Marcel A. G. van der Heyden

**Affiliations:** Department of Medical Physiology, Division of Heart & Lungs, University Medical Center Utrecht, Yalelaan 50, 3584 CM Utrecht, The Netherlands

**Keywords:** K_IR_2.1, AgoKir, long-term effects, propafenone, trafficking

## Abstract

Expression and activity of inwardly rectifying potassium (K_IR_) channels within the heart are strictly regulated. K_IR_ channels have an important role in shaping cardiac action potentials, having a limited conductance at depolarized potentials but contributing to the final stage of repolarization and resting membrane stability. Impaired K_IR_2.1 function causes Andersen-Tawil Syndrome (ATS) and is associated with heart failure. Restoring K_IR_2.1 function by agonists of K_IR_2.1 (AgoKirs) would be beneficial. The class 1c antiarrhythmic drug propafenone is identified as an AgoKir; however, its long-term effects on K_IR_2.1 protein expression, subcellular localization, and function are unknown. Propafenone’s long-term effect on K_IR_2.1 expression and its underlying mechanisms in vitro were investigated. K_IR_2.1-carried currents were measured by single-cell patch-clamp electrophysiology. K_IR_2.1 protein expression levels were determined by Western blot analysis, whereas conventional immunofluorescence and advanced live-imaging microscopy were used to assess the subcellular localization of K_IR_2.1 proteins. Acute propafenone treatment at low concentrations supports the ability of propafenone to function as an AgoKir without disturbing K_IR_2.1 protein handling. Chronic propafenone treatment (at 25–100 times higher concentrations than in the acute treatment) increases K_IR_2.1 protein expression and K_IR_2.1 current densities in vitro, which are potentially associated with pre-lysosomal trafficking inhibition.

## 1. Introduction

Inward rectification was first detected in 1949 by Bernard Katz [1]. At first, it was called “anomalous rectification” to distinguish it from voltage-gated K (Kv) channel current in squid giant axons [2,3]. Since then, significant progress has been made in this area of research. These developments include cloning the members of inward rectifier channels, discovering the molecular mechanism of inward rectification, implementing genetic studies in experimental animals, constructing mutations in inward rectifier genes, and so on [4,5,6,7,8,9,10]. The K_IR_2.1 channel protein is encoded by *KCNJ2,* and K_IR_2.1 current is a major part of the inward rectifying potassium current (IK_1_) in cardiomyocytes, as no detectable current was observed in ventricular myocytes from K_IR_2.1 knockout mice [2,11,12]. IK_1_ generated by K_IR_2.x channels (K_IR_2.1, K_IR_2.2, and K_IR_2.3 homo- and heterotetramers) is responsible for controlling the resting membrane potential and accelerating the final repolarization phase in cardiomyocytes [2,13]. Therefore, regulating the IK_1_ current can greatly affect the excitability and arrhythmogenesis of cardiomyocytes [13,14]. Recent findings and the development of new compounds may yield new pharmacological IK_1_ modulators [10,15,16,17].

The strong inward rectification of K_IR_2.1 channels mainly results from the interaction with intracellular polyamines as they block the outflux of K^+^ by binding to the negatively charged residues in the transmembrane (D172) and cytoplasmic domain (E224, E229) of the channel [2,4,6,7,13,18,19,20,21,22]. Gain-of-function in K_IR_2.1 can cause atrial fibrillation [14,23]. On the contrary, dysfunction of K_IR_2.1 can prolong the duration of the cardiac action potential, which can cause Andersen-Tawil Syndrome (ATS) [14,24,25,26,27]. Furthermore, heart failure is associated with decreased IK_1_ density [28]. For the latter situations, restoration of normal K_IR_2.1 function by agonists of K_IR_2.1 (AgoKirs) would be beneficial [29].

The drug propafenone—an orally active sodium channel blocking agent—was identified as an AgoKir [29,30]. It is currently used as a class Ic antiarrhythmic drug [31,32]. Previous experiments revealed that low concentrations (0.5–1 µM) of propafenone acutely increase IK_1_ by binding with Cys311 of the K_IR_2.1 protein [29,30,31,33]. Since the effect of propafenone on IK_1_ generated by K_IR_2.1 is acute, it implies that chronic treatment will be needed in patients to obtain a permanent benefit. It has been established that drugs influencing ion channel activity directly could also alter ion channel expression and function in the long-term [34,35]. However, the long-term influence of propafenone on K_IR_2.1 channels is unknown. Therefore, we explored whether propafenone affects K_IR_2.1 expression, its subcellular localization, and its functional consequence in the long-term.

In the current work, we confirm that propafenone can act as an AgoKir. We explored the long-term effects of propafenone on IK_1_ and K_IR_2.1, but also its close homologue K_IR_2.2, channel expressions. Live imaging was also performed to determine the subcellular localization of K_IR_2.1 proteins. As K_IR_2.1 channel proteins degrade via the lysosomal pathway [36], the half-life of the protein in the presence of propafenone was determined [36,37,38]. This study provides a basis for further research on propafenone-based AgoKirs, thus contributing to the development of a therapy for diseases in which the function of K_IR_2.1 is reduced.

## 2. Results

### 2.1. As an AgoKir, Propafenone Can Increase Both Channel Expression and IK_1_ Density

To confirm that propafenone could increase K_IR_2.1 channel generated IK_1,_ the acute effects of low doses of propafenone on IK_1_ were investigated by whole-cell patch-clamping in HEK-KWGF cells. Analyses were performed separately for each measured voltage point. Statistical analysis showed that the outward component of (−60 to −20 mV) IK_1_ was increased, especially at −40 mV, when compared with the control upon perfusion with 1 µM propafenone (Figure S1). Whereas, at a concentration of 25 μM, both the inward and outward components are decreased (Figure S1). Similar results had been found previously [30].

**Figure 1:**
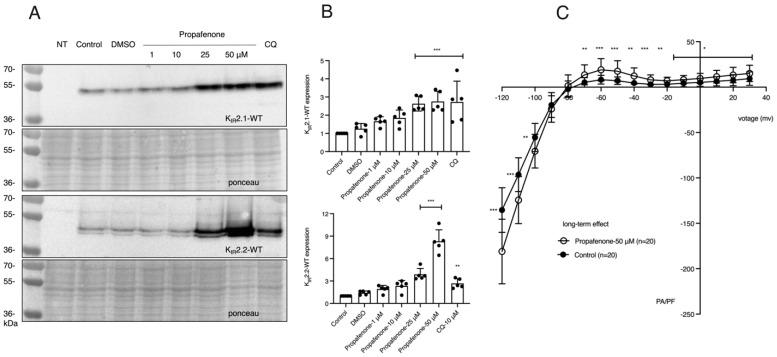
24 h effects of propafenone on K_IR_2.x expression and function. (**A**) Western blot analysis of human WT K_IR_2.1 and K_IR_2.2 expression in HEK-293 cells. Cells were treated with different concentrations of propafenone (1, 10, 25, 50 µM) for 24 h (*n* = 5). Non-transfected cells (NT) were used as a negative control. (**B**) Summarized results of K_IR_2.1 and K_IR_2.2 expression in control and cells treated for propafenone (*n* = 5). The control protein level was set at 1 after correction for loading. Ponceau staining was used as a loading control. (**C**) Propafenone treatment increased both inward and outward I_KIR2.1_ in HEK-KWGF cells. Current-voltage relationship of mean I_KIR2.1_ current values ± SD, measured in the absence of propafenone. In the treatment group, cells were pretreated with propafenone for 24 h with a final concentration of 50 µM. The control group did not undergo any treatment. *n* = 20 cells for each group. Lysosomal inhibitor chloroquine, CQ, is used as a positive control. * *p* < 0.05, ** *p* < 0.01, *** *p* < 0.001 vs. control.

Next, the long-term effects of propafenone on IK_1_ and K_IR_2.1 and K_IR_2.2 protein expression were explored. Figure 1A,B show that propafenone can increase the expression levels of K_IR_2.1 and K_IR_2.2 proteins dose-dependently. To see whether the increased protein levels observed by western blot would also result in increased expression levels on the membrane and K_IR_2.1-carried current, we treated HEK-KWGF cells with 50 μM propafenone for 24 h and analyzed them immediately for IK_1_ current densities in the absence of propafenone (Figure 1C). Long-term treatment of the cells with propafenone significantly increased both the inward component of IK_1_ at membrane potentials between −120 and −100 mV and the outward component at membrane potentials between −70 and 30 mV.

### 2.2. Propafenone Specifically Works on K_IR_2.1 Channels and Shows a Long Residence Time

**Figure 2:**
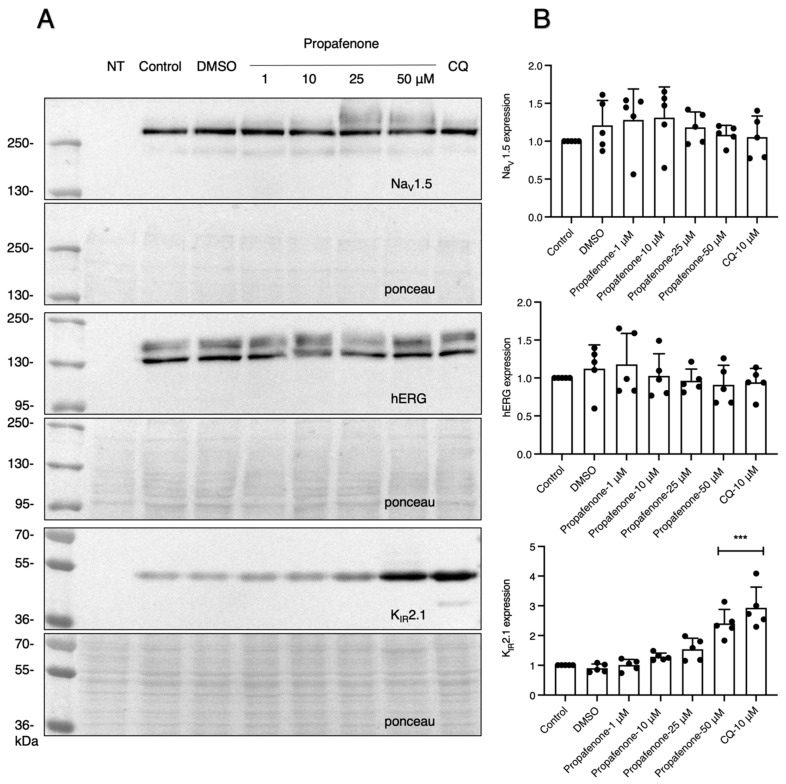
Propafenone specificity for K_IR_ channels. (**A**) Western blot analysis of hERG channel protein, sodium channel protein, and K_IR_2.1 channel protein expression levels. K_v_11.1 is a voltage-activated potassium channel expressed as a core N-glycosylated immature form (~135 kDa) and a fully N-glycosylated mature form (~155 kDa) in HEK-hERG cells [39]. Na_v_1.5 is a voltage-activated sodium channel expressed as a single band (~250 kDa) in the HEK-Na_v_1.5 cells [40]. Cells were treated with different concentrations of propafenone (1, 10, 25, and 50 µM) for 24 h (*n* = 5). Non-transfected cells (NT) were used as a negative control. (**B**) Summarized results of (**A**) (*n* = 5). The control protein level was set at 1 after correction for loading. Ponceau staining was used as a loading control. *** *p* < 0.001 vs. control.

After determining the long-term effect of propafenone on K_IR_2.1 channel expression, we next investigated hERG (K_v_11.1) and sodium channels (Na_v_1.5) to test if propafenone has a similar effect on other channel protein types over the same time period. HEK-hERG cells and HEK-Na_v_1.5 cells were used. As the HEK-Na_v_1.5 cell line stably expresses both Na_v_1.5 and K_IR_2.1 channels, we determined Na_v_1.5 protein expression alongside K_IR_2.1 expression levels from the same samples. As shown in Figure 2A,B, the expression levels of Na_v_1.5 and K_v_11.1 channel proteins did not change in response to propafenone treatment. The expression level of K_IR_2.1 protein was increased similarly, as shown in Figure 1A, which indicates that propafenone can specifically work on K_IR_ channels, even in the presence of another ectopically expressed ion channel (i.e., Na_v_1.5).

**Figure 3:**
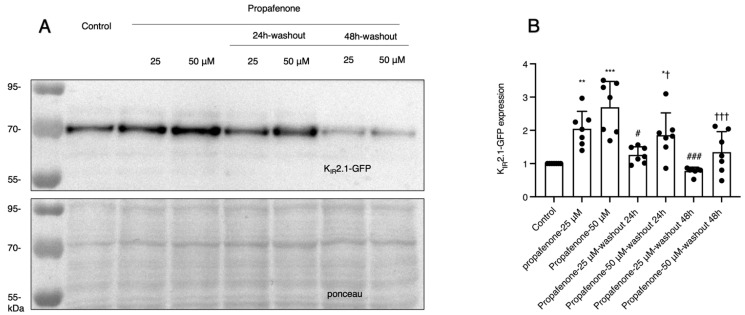
Washout effect of propafenone on K_IR_2.1 expression. (**A**) K_IR_2.1 expression before and after washout (*n* = 7). (**B**) Summarized results of (**A**). The control protein level was set at 1 after correction for loading. Ponceau staining was used as a loading control. * *p* < 0.05, ** *p* < 0.01, *** *p* < 0.001 vs. control; # *p* < 0.05, ### *p* < 0.001 vs. propafenone-25 µM group; † *p* < 0.05, ††† *p* < 0.001 vs. propafenone 50 µM group.

To determine the retention time of propafenone’s effect on K_IR_2.1 expression levels following drug removal, a washout experiment was performed. The expression levels of K_IR_2.1 proteins decreased significantly after washout, showing propafenone’s washout effect (Figure 3A,B). However, for the cells that received treatment with 50 µM propafenone, the expression of K_IR_2.1 remained high after washout for 24 h (Figure 3A,B). This long residence time (24 h) indicates the persistence of propafenone’s chronic effect.

### 2.3. Channel Function, Polyamine Binding Sites, and the Drug-Channel Interaction Location Do Not Interfere with the Long-Term Effect of Propafenone on K_IR_2.1 Expression

**Figure 4:**
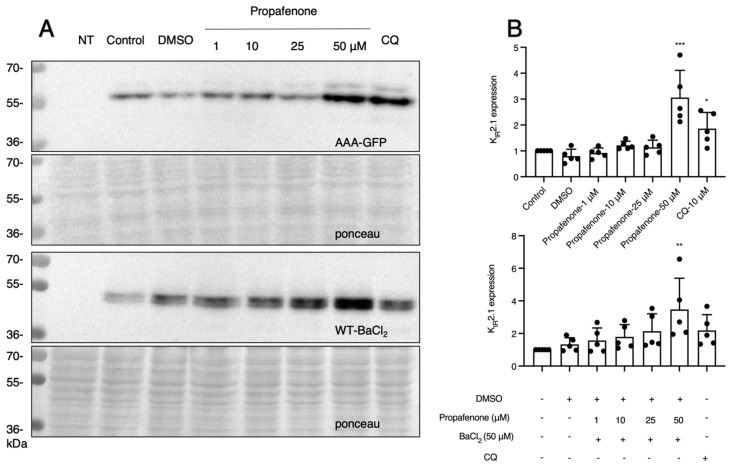
Channel function does not affect the changes in expression of K_IR_2.1 proteins in response to propafenone. (**A**) K_IR_2.1 expression of cells after being treated with different concentrations of propafenone (1, 10, 25, and 50 µM) for 24 h (*n* = 5). (**B**) Summarized results of K_IR_2.1 expression in control and cells treated for propafenone (*n* = 5). The control protein level was set at 1 after correction for loading. Non-transfected cells (NT) were used as a negative control. Ponceau staining was used as a loading control. * *p* < 0.05, ** *p* < 0.01, *** *p* < 0.001 vs. control of propafenone.

**Figure 5:**
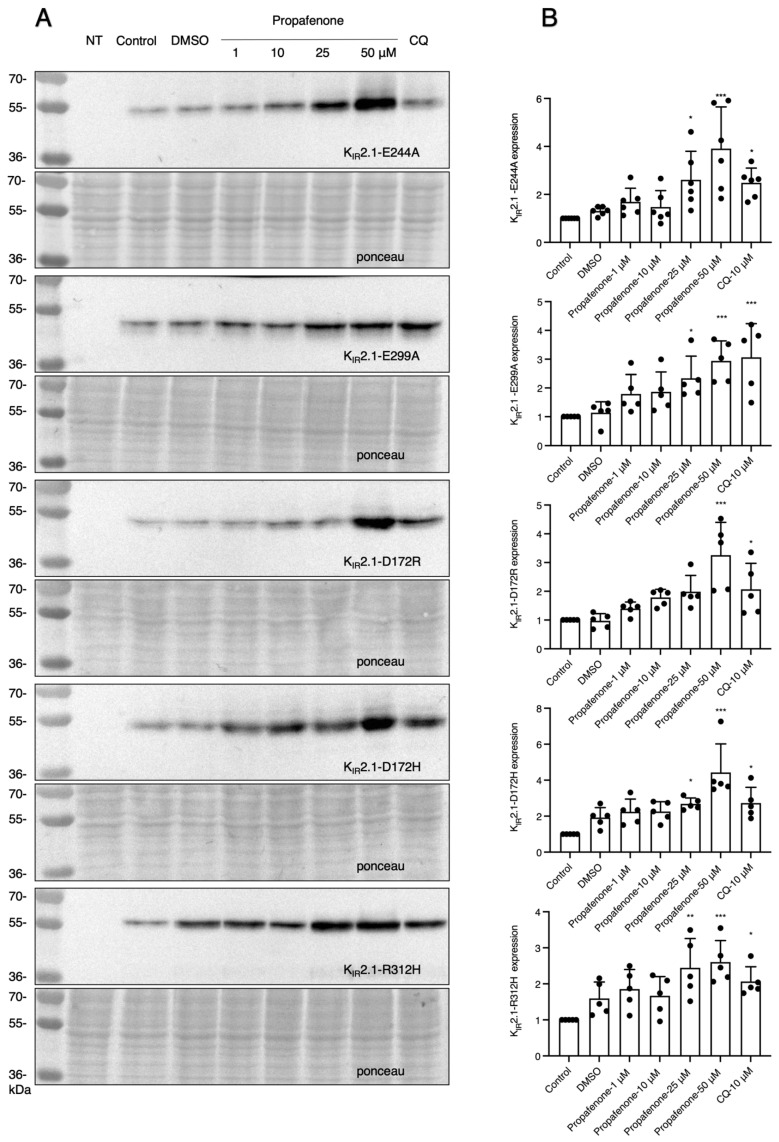
Mutations of polyamine binding sites and the drug-channel binding location do not interfere with the chronic effect of propafenone on K_IR_2.1 protein expression. (**A**) K_IR_2.1 expression of cells after treatment with increasing concentrations of propafenone in different mutant K_IR_2.1 proteins (D172R (*n* = 5), D172H (*n* = 5), E244A (*n* = 6), E299A (*n* = 5), and R312H (*n* = 5)). Cells were treated with different concentrations of propafenone (1, 10, 25, 50 and µM) for 24 h. (**B**) Summarized results of K_IR_2.1 expression levels in control and cells treated for propafenone (*n* = 5 or 6). The control protein level was set at 1 after correction for loading. Non-transfected cells (NT) were used as a negative control. Ponceau staining was used as a loading control. * *p* < 0.05, ** *p* < 0.01, *** *p* < 0.001 vs. control.

As IK_1_ current plays an important role in regulating various physiological processes, it is essential to know whether channel functions were involved in the long-term effect of propafenone. We used a K_IR_2.1-AAA non-conducting channel protein. In addition, BaCl_2_ was used as a channel-blocking agent. It demonstrated that the expression of K_IR_2.1 proteins was increased similarly as compared to WT at higher concentrations of propafenone (50 µM), while IK_1_ was inactivated by the K_IR_2.1-AAA mutation or blocked by BaCl_2_ (Figure 4A,B). Since polyamine binding sites (D172, E299, and E244) play important roles in the inward rectification of K_IR_2.1 channels and direct drug-channel interference is indispensable in the acute reactions of propafenone [2,29], we further investigated the roles of these factors. Western blots showed similar effects for these mutant proteins when compared with WT (Figure 5).

### 2.4. K_IR_2.1-GFP/Dendra2 Clustering and Protein Turnover Rate Indicate That Propafenone May Interfere in Late Endosome Function

**Figure 6:**
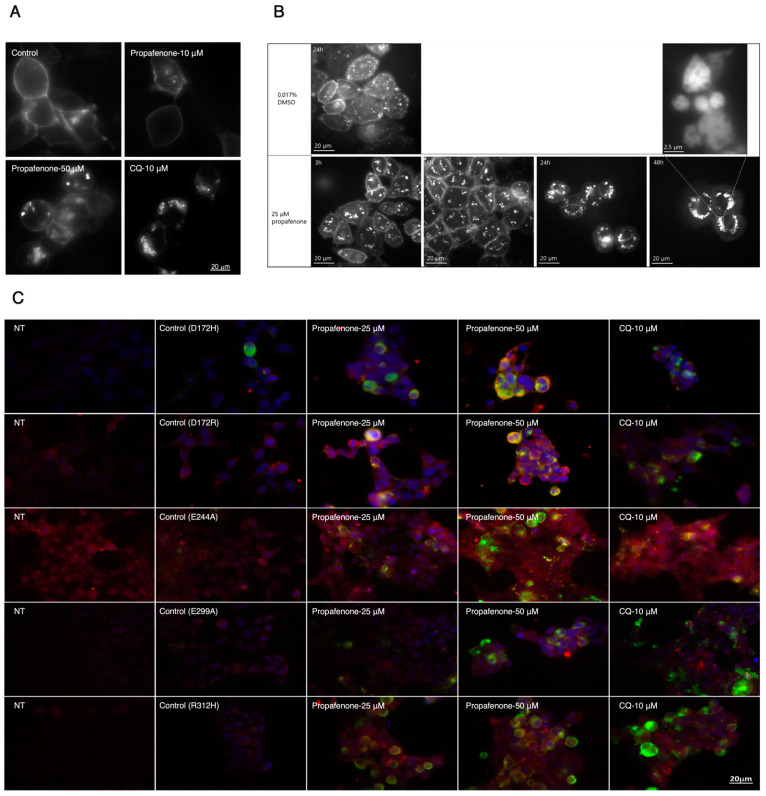
Propafenone induces K_IR_2.1 protein clustering in HEK-KWGF cells, CHO-KD cells, and HEK293 cells. (**A**) Propafenone (10, 50 µM, 24 h) induced intracellular accumulation of WT and mutant K_IR_2.1 proteins in HEK-KWGF cells. 488 nm laser light was used to visualize K_IR_2.1-GFP. The scale bar indicates 20 µm. (**B**) Cloned CHO-KD cells were incubated for 24 h with DMSO or propafenone at 25 µM for different periods (3, 6, 24, or 48 h). Images were taken at 60 x magnification. 488 nm laser light was used to visualize K_IR_2.1-Dendra2. The scale bar indicates 20 µm. (**C**) Subcellular localization of D172R, D172H, E244A, E299A, and R312H K_IR_2.1 proteins in HEK293 cells. Cells were treated with different concentrations of propafenone (25, 50 µM) for 24 h. Non-transfected cells (NT) were used as a negative control. Propafenone and CQ treatment induce K_IR_2.1 protein accumulation. K_IR_2.1 was detected by specific antibodies (green) Cadherin (membrane staining) by Pan-Cadherin antibodies (red). The nuclei were stained with DAPI (blue). The scale bar indicates 20 µm.

Live imaging showed that propafenone and chloroquine (CQ) can both cause intracellular K_IR_2.1 accumulation but in a different pattern (Figure 6A). CQ causes scattered clusters of K_IR_2.1 on the edge of the cell, while propafenone causes brighter clusters both in the edge and center of the cell at 50 μM, but not at 10 μM. As shown before, CQ increases the expression of K_IR_2.1 protein by inhibiting its degradation via the lysosomal pathway [36]. The different appearance of clusters in response to propafenone may point to a different mechanism for cell trafficking in comparison to CQ.

CHO-KD cells were used to investigate whether propafenone’s long-term effect is late endosome/lysosome-related. K_IR_2.1-Dendra2 is present in round clusters in the interior of the cells, which accumulate inside cells upon administration of 25 μM propafenone (3–48 h) (Figure 6B). Images of DMSO-treated cells (Figure 6B) show that K_IR_2.1-Dendra2 is present in the plasma membrane and the cell’s interior as many little clusters. Time-lapse imaging (Video S1, CHO-KD cells treated with DMSO) shows that these clusters move fast in all directions. K_IR_2.1-Dendra2 moves slower after being treated with propafenone, and big clusters appeared in the cells’ interiors (Video S1). The number of small clusters decreased (Video S1, CHO-KD cells treated with 25 µM propafenone for 3 h, 6 h, 24 h, and 48 h), and more and larger clusters became visible in the cells. Video S1 shows that the larger clusters of K_IR_2.1-Dendra2 display less movement, while the remaining small clusters move faster. For the cells treated for 48 h with propafenone, multivesicular bodies (MVBs) appeared among the clusters of proteins (Figure 6B, enlarged picture), indicating that K_IR_2.1-Dendra2 might accumulate in late endosome-like structures. Similarly, as seen for protein expression levels (Figure 5), intracellular K_IR_2.1 protein accumulation was independent of D172H, D172R, E244A, E299A, or R312H mutations (Figure 6C).

**Figure 7:**
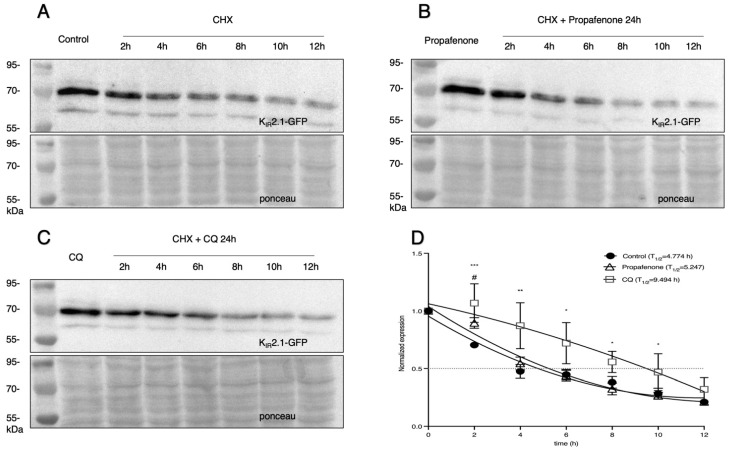
Cycloheximide (CHX) assay of K_IR_2.1 degradation in HEK-KWGF cells. (**A**) Example of K_IR_2.1 protein degradation after exposure to 200 µg/mL CHX for different time intervals. (**B**) Example of K_IR_2.1 protein degradation in cells that were treated with propafenone for 24 h. 200 µg/mL CHX was added for different time periods before lysates were prepared at t = 24 h. (**C**) Example of K_IR_2.1 protein degradation in cells that were treated with CQ for 24 h. 200 µg/mL CHX was added for different time periods before lysates were prepared at t = 24 h. (**D**) Quantification of CHX assays to depict normalized K_IR_2.1 expression vs. timed CHX treatment with or without propafenone or CQ treatment (*n* = 5). The dotted line indicates 50% of the initial normalized K_IR_2.1 protein signal. * indicates *p* < 0.05, ** indicates *p* < 0.01 *** indicates *p* < 0.001 when CQ vs. control. # indicates *p* < 0.05 when comparing propafenone vs. control. To show the declining trend of protein expression after exposure to 200 µg/mL CHX, the loading protein in Figure (**A**) was twice that of Figure (**B**) and Figure (**C**).

MVBs will deliver cargo destined for degradation to the lysosome [41,42]. Therefore, we tested the half-life of the K_IR_2.1 proteins in HEK-KWGF cells in the presence of the translation inhibitor cycloheximide (CHX) (200 µg/mL). In the CQ treated group, the T_1/2_ was significantly increased compared to the control (T_1/2_ = 9.494 h vs. 4.774 h, Figure 7C,D). In contrast, no significant difference in T_1/2_ was found following propafenone treatment (T_1/2_ of 4.774 h in the control group vs. 5.247 h after propafenone treatment) (Figure 7A,B). Thus, propafenone does not impair the degradation of K_IR_2.1 proteins in lysosomes.

## 3. Discussion

We confirmed that acute administration of propafenone at low concentrations increases K_IR_2.1 currents in HEK-KWGF cells, which is similar to the results obtained in CHO cells transiently transfected with WT K_IR_2.1 [30]. Therefore, propafenone was shown to act as a K_IR_2.1 agonist, which we named “Agokir” [29]. However, since the effect of propafenone on K_IR_2.1 carried current is acute, chronic treatment will be required for long-term IK_1_ enhancement. Therefore, we investigated propafenone’s long-term effect on K_IR_2.1 channels.

Propafenone is commonly administered in the clinic to treat atrial fibrillation because of its sodium channel blocking activity [43,44]. This sodium channel blocking property results in a markedly depressed depolarization phase of the action potential and a widening of the QRS complex [45,46,47]. Some studies also showed that QRS duration was increased with or without QT interval prolongation in humans after treatment with propafenone [48,49,50,51,52]. Therapeutic plasma levels of propafenone in humans were estimated to range from 0.53 to 5.28 µM [33,53,54]. Furthermore, propafenone concentrations in human atrial tissues were on average ten times higher than those found in the plasma [53]. Such concentrations approach or are even similar to the concentrations found in our work, in which the chronic effect on K_IR_2.1 protein results in an increase in expression levels.

In the present work, propafenone increases the outward component acutely at low concentrations (0.5 and 1 μM). Such an increase was shown to be achieved by the propafenone-K_IR_2.1 channel binding-mediated decrease of channel affinity for polyamines and thus current rectification [30]. In contrast, propafenone at higher concentrations (25 and 50 μM) shows a strong acute blocking effect on both the inward and outward components. This block is caused by a propafenone-mediated decrease of the negative charge of the channel pore and channel affinity for phosphatidylinositol 4,5-bisphosphate (PIP_2_), which is a lipid critical for channel activation [12,43]. For the long-term effect, however, propafenone treatment at high concentrations results in a significant increase in IK_1_ densities. These latter electrophysiological measurements were performed in the absence of propafenone, thereby excluding its acute effect on K_IR_2.1 channels. This significant increase likely occurred because propafenone inhibits channel backward trafficking, thereby indirectly increasing the K_IR_2.1 channel expression level on the cell membrane.

In order to test propafenone’s specificity for increasing K_IR_2.1 channel expression, we investigated its effects on hERG channel expression, which is a voltage-gated potassium channel and thereby different from the non-voltage-gated K_IR_2.1 channel. Sodium channel expression was also tested because propafenone is used as its blocking agent in the clinic [33,55]. Propafenone did not interfere with the expression levels of these two channels, revealing that propafenone has at least some specificity towards K_IR_ channels. Some studies showed that there is reciprocal regulation between Na_v_1.5 and K_IR_2.1 channels [56,57,58,59,60,61,62]. K_IR_2.1 overexpression increases the expression levels of Na_v_1.5 in mouse hearts [58]. As propafenone increases the expression level of K_IR_2.1 proteins significantly but does not interfere with that of Na_v_1.5 in the same cells and with the same treatment in our study. We may thus hypothesize that propafenone might either affect cooperation between the two channels or act on a part of the trafficking pathway in which both channels do not cooperate. The previous study also showed that Na_v_1.5 protein reduces K_IR_2.1 protein internalization and promote its presence at the cell surface [58]. As the expression level of Na_v_1.5 was not changed, the increased expression of K_IR_2.1 protein was only affected by propafenone.

Ba^2+^ is an efficient IK_1_ blocking ion [10,63]. The atomic radius of Ba^2+^ is close to that of K^+^; therefore, it will fit into the K^+^ selectivity filter and remain in that position due to its larger charge, effectively blocking the inward and outward K^+^ flow [64,65,66]. At the same time, we also investigated a non-conducting K_IR_2.1-AAA mutant to test the influence of channel functions on the long-term effect of propafenone. The results showed that the expression of K_IR_2.1 channel proteins displayed no significant differences when compared with WT or not inhibited channels, which suggests that channel function (i.e., K^+^ conduction) is not involved in the long-term effect of propafenone.

Polyamines, responsible for naturally occurring inward rectification, occupy two positions in the K_IR_2.x channels: the cytoplasmic pore domain at K_IR_2.1 E224 and E299 and the transmembrane pore domain at D172 [2,22]. Western blotting pointed out that propafenone dose-dependently increases WT, E224A, E299A, D172H, and D172R-K_IR_2.1 protein expression in HEK-293 cells. Therefore, polyamine binding sites, and most likely polyamine binding too, appear not to be involved in the propafenone-mediated increase in K_IR_2.1 expression levels.

Dynamics simulations predicted that propafenone interacts with K_IR_2.1 by forming a hydrogen bond with the cysteine residue C311, which is identified as a direct channel-drug binding site [29,30,67,68]. Because of the proximity of C311 to the R312 residue [69], it is possible that the mutation R312H allosterically modifies the 310-QCRSSY-315 C-terminus domain, thereby precluding propafenone channel interaction. Increased K_IR_2.1-R312H expression showed a similar result as WT, revealing that drug-channel interaction is most likely not involved in the chronic response to propafenone. In conclusion, propafenone specifically works on K_IR_2 channels, but neither K^+^ conduction, polyamine binding sites, nor direct drug-channel interactions are involved in the long-term effects of propafenone.

HEK-KWGF cells showed an intracellular accumulation of K_IR_2.1 proteins after being treated with propafenone. The intracellular patterns, however (Figure 6A), were distinct from cells treated with CQ, which is known to inhibit lysosomes. A potential explanation for this difference is that propafenone acts on both late endosomes and lysosomes, or on endosomes only. Live imaging on CHO-KD cells supported the observations reported above and revealed that after incubating 25 µM of propafenone for only 3 h, K_IR_2.1-Dendra2 proteins started to accumulate in the cytoplasm (Figure 6B). More and bigger clusters appeared at the following time points. The large clusters of K_IR_2.1-Dendra2 proteins show less movement, which is in line with earlier research in which lysosomal diameter was increased using sucrose; enlarged lysosomes were correlated to a reduced diffusion rate [70]. Furthermore, MVBs were shown in the protein clusters after 48 h of incubation with propafenone, indicating that K_IR_2.1-Dendra2 may accumulate within the late endosome compartment [71]. Protein also accumulates in late endosomes when lysosomes do not function well [71,72]. However, propafenone treatment did not increase T_1/2_ as CQ, indicating that propafenone does not inhibit the function of lysosomes per se. From all these results, we conclude that chronic propafenone treatment increases K_IR_2.1 protein expression and K_IR_2.1 current densities in vitro following a 24 h treatment, which persists after washout and is potentially associated with pre-lysosomal trafficking inhibition. Moreover, additional proteins following a similar degradation route as K_IR_2.1 could be affected similarly to propafenone.

Our data shows that propafenone can function as AgoKir at low concentrations without disturbing K_IR_2.1 protein handling; however, it also shows long-term effects at higher concentrations. It is worthwhile to search for more potent propafenone analogs that should increase IK_1_ without affecting K_IR_2.1 channel expression, thus contributing to the development of new therapeutic avenues to address diseases related to dysfunctional K_IR_2.1.

## 4. Materials and Methods

### 4.1. Cell Culture

Cell lines were cultured in Dulbecco’s Modified Eagles Medium (DMEM; Lonza, Breda, The Netherlands) supplemented with 10% fetal bovine serum (FBS; Sigma-Aldrich, St. Louis, MO, USA), 200 mM L-glutamine (Lonza), and 10.000 U/mL penicillin-streptomycin (Lonza) at 37 °C with 5% CO_2_. These cells contain human embryonic kidney (HEK)-293 cells (ATCC, Accession Number: CRL-1573), HEK-KWGF cells [73] (HEK cells stably expressing C-terminal GFP-tagged murine K_IR_2.1), CHO-KD cells [74] (Chinese Hamster Ovary cells stably expressing Dendra-2-tagged K_IR_2.1), HEK-hERG cells [75] (cells stably expressing human hERG), and HEK-Na_v_1.5 cells [75] (HEK cells stably expressing both K_IR_2.1 and human sodium channels (Na_v_1.5)). Cells for each time point were seeded on the same day, and drugs were added for the indicated time before the harvest of all samples. Cell confluency at the time of processing was 80–90%, 50–60%, and 10% for biochemical, (immuno)fluorescent, and patch-clamp electrophysiology experiments, respectively.

### 4.2. K_IR_2.1-Mutant Expression Constructs and Transfection

K_IR_2.1-E224A and E299A mutant constructs were obtained from Dr. Tristani-Firouzi (University of Utah School of Medicine, USA), and functional characteristics have been described previously by others [76] and us [34,77]. K_IR_2.1-D172R and D172H mutant constructs were obtained from Dr. So (Seoul National University, College of Medicine, Republic of Korea), and functional characteristics have been described before [78]. A K_IR_2.1-R312H mutant construct was obtained from Dr. Bendahhou (Université Côte d’Azur, France), and functional characteristics have been described recently [79]. Cell transfection was performed with linear polyethylenimine (PEI) with a molecular weight of 25,000 (Polysciences, Hirschberg an der Bergstrasse, Germany) as described previously by us [80] and references therein. Transfection efficacies were 30–70% and routinely checked by GFP transfection.

### 4.3. Drugs

Chloroquine (CQ) (Sigma, St. Louis, MO, United States, cat. No. C6628) was dissolved in sterile water at a concentration of 10 mM and stored at −20 °C. Propafenone (Sigma, cat. No. C7698) was dissolved in DMSO at a concentration of 100 mM and stored at −20 °C until use. Cycloheximide (CHX, Sigma, cat. No. C7698) was dissolved in sterile water at a concentration of 5 mg/mL, stored, and aliquoted at −20 °C until use. All drugs were diluted on the day they were used.

### 4.4. Western Blot

Cell lysates were prepared in Buffer D (20 mM HEPES, 125 mM NaCl, 10% glycerol, 1 mM EGTA, 1 mM dithiothreitol, 1 mM EDTA, and 1% Triton X-100 (pH 7.6)) supplemented with 0.2 mM phenylmethylsulfonyl fluoride (PMSF) and 4 μg·mL^−1^ aprotinin. Protein lysate (30 or 60 μg) was separated by 7% or 10% SDS-PAGE and blotted onto a nitrocellulose membrane (Bio-Rad Laboratories, Veenendaal, The Netherlands). Ponceau staining was used as a loading control for subsequent quantification. Blots were blocked for 1 h with 5% Protifar dissolved in Tris-buffered saline/Tween 20 (20 mM Tris-HCl (pH 8.0), 150 mM NaCl, 0.05% (*v*/*v*) Tween-20). For protein detection, the membrane was incubated with anti-K_IR_2.1 (1:1000; Sigma-Aldrich, St. Louis, MO, USA), anti-GFP (1:500; Santa Cruz Biotechnology, Heidelberg, Germany), anti-hERG (1:2500; Alomone Labs, Jerusalem, Israel), or anti-sodium channel primary antibody (1:2000; custom-made [81]). A peroxidase-conjugated Goat anti-Mouse (Jackson ImmunoResearch, West Grove, PA, USA) or Goat anti-Rabbit (Jackson ImmunoResearch, West Grove, PA, USA) antibody was applied as the second primary antibody. Final detection was performed using the Standard ECL procedure (Amersham Bioscience, Buckinghamshire, UK). Quantification was done by Image Lab software version 6.1 (Bio-Rad Laboratories, Veenendaal, The Netherlands).

### 4.5. Cloning

CHO-KD single-cell suspension was counted in a Brand™ Bürker counting chamber (Fisher Scientific, Landsmeer, The Netherlands). The cell suspension was diluted to obtain a concentration of 10 cells per mL, and cells were cultured in 96-well plates (100 μL/well). Cells were examined under a Nikon TMS inverted microscope (Nikon Instruments Europe B.V., Amsterdam, The Netherlands) after forming a single clone. The clones were expanded and then imaged by a Nikon Eclipse 80i epifluorescence microscope (Nikon Instruments Europe B.V.).

### 4.6. Live Imaging with Confocal Microscopy

HEK-KWGF cells were treated with propafenone (10, 50 µM) for 24 h, and then 488 nm laser light was used to visualize K_IR_2.1-GFP. We cloned the existing CHO-KD cell line to create a pool of cells with high K_IR_2.1-Dendra2 expression. CHO-KD cells were treated with propafenone at 25 µM for different time courses (3, 6, 24, and 48 h). Then they were placed under a Nikon Eclipse Ti2-E inverted microscope (Nikon Instruments Europe B.V.) equipped with a ×60 oil immersion objective (numerical aperture 1.49; CAIRN research, Faversham, United Kingdom) at room temperature. The laser light at 488 nm was used to visualize K_IR_2.1–Dendra2. For 20 min, a photo was taken every 20 s. Movie Maker (Microsoft, 2012) was used to make a time-lapse of 60 images.

### 4.7. Immunofluorescence Microscopy

HEK-293 cells were cultured on Ø 15 mm glass coverslips coated with 0.1% gelatin. Different K_IR_2.1 mutant expression constructs (E224A, E299A, D172R, D172H, and R312H) were transfected into HEK-293 for 24 h using PEI before the cells were treated with propafenone (25, 50 µM) or CQ (10 µM) for 24 h. Then the coverslips were washed with PBS, fixated with 3%-paraformaldehyde, permeabilized with 0.5% Triton X-100 (in PBS), quenched with PBS/glycine (50 mM), and incubated twice with NET-gel (0.25% gelatin, 50 mM Tris-Cl, 150 mM NaCl, 4 mM EDTA, 0.05% Igepal, ± 0.01% NaN_3_, pH 7.4). Primary antibodies used were K_IR_2.1 (1:400; Sigma-Aldrich, St. Louis, MO, USA) and Pan-Cadherin (1:800; Sigma-Aldrich, St. Louis, MO, USA). After the cells were washed, they were incubated with secondary antibodies: goat anti-mouse (Green, 1:100; Jackson ImmunoResearch Laboratories Inc., West Grove, PA, USA) and donkey anti-rabbit (Alexa Red, 1:350; Jackson ImmunoResearch Laboratories Inc.). Cell nuclei were stained with 4′,6-diamidino-2-phenylindole (DAPI; 1:100; Molecular Probes, Leiden, The Netherlands). Coverslips were mounted with Vectashield (Vector Laboratories Inc., Burlingame, CA, USA) and imaged using a Nikon Eclipse 80i (Nikon, Amsterdam, The Netherlands) and NIS elements Basic Research (Nikon, Amsterdam, The Netherlands) software.

### 4.8. Washout Experiment

HEK-KWGF cells were seeded in Ø 60 mm dishes overnight. 24 h after treatment with propafenone (25 or 50 μM), the medium of the cells was replaced by fresh supplemented DMEM. Protein lysates were harvested 24 or 48 h after changing the medium. The K_IR_2.1 expression level was detected by Western blot.

### 4.9. Cycloheximide Assay

HEK-KWGF cells were seeded in Ø 35 mm dishes. After 24 h, cells were left untreated (control) or treated with 50 μM propafenone or 10 μM CQ for 24 h. 200 μg/mL cycloheximide was added during the last phases (2, 4, 6, 8, 10, or 12 h) of the 24 h treatment period. Cell lysates were prepared and processed as indicated in Section 4.4. Since propafenone and CQ increased the expression levels of K_IR_2.1-GFP protein compared to control conditions before the start of CHX application, twice the amount of control lysate loaded on SDS-PAGE to enable visualization of the K_IR_2.1 protein under control conditions at CHX t = 10 and t = 12 using similar ECL exposure times as for propafenone and CQ conditions.

### 4.10. Patch Clamp Electrophysiology

Whole-cell clamp measurements were performed using an AxoPatch 200B amplifier controlled by pClamp10.4 software (Molecular Devices, LLC, San Jose, CA, USA). The K_IR_2.1 current in HEK-KWGF cells was measured at room temperature. Patch pipettes were made with a Sutter P-2000 puller (HEKA Elektronik, Lambrecht, Germany) and had resistances of 1–3 MΩ.

HEK-KWGF cells were grown on 0.1% gelatin (Bio-Rad, Veenendaal, The Netherlands), coated Ø 12-mm coverslips in a 12-well plate. For acute effect, after taking baseline measurements, the cells were perfused for 5 min with propafenone (0.5, 1, and 25 µM), followed by a 5-min washout. For the chronic effect, cells were randomly divided into two groups, the treatment groups were treated with 50 µM of propafenone for 24 h, while the control group did not undergo any treatment. To record K_IR_2.1 currents, voltage-clamp measurements were performed by applying 1s test pulses ranging between −120 and +30 mV with 10 mV increments. Extracellular solution for whole-cell I_KIR2.1_ measurements contained (in mmol/L): NaCl 140, KCl 5.4, CaCl_2_ 1, MgCl_2_ 1, glucose 6, NaHCO_3_ 17.5, HEPES 15, pH 7.4/NaOH. The pipette solution contained (in mmol/L) potassium gluconate 125, KCl 10, HEPES 5, EGTA 5, MgCl_2_ 2, CaCl_2_ 0.6, Na2ATP 4, pH 7.20/KOH.

### 4.11. Statistics

Data are expressed as mean ± S.D. Differences between group averages were tested using a one-way ANOVA with a post-hoc test (Tukey’s HSD), or an unpaired *T*-test. Data were considered significant when the *p*-value was less than 0.05. Statistical analysis was performed using GraphPad Prism version 9 (GraphPad Software, San Diego, CA, USA).

## Data Availability

All the research data are available in article and the supporting information section.

## Supplementary Materials

The following supporting information can be downloaded at: https://www.mdpi.com/article/10.3390/ph16030404/s1, Figure S1: Acute effect of propafenone; Video S1: Propafenone time-dependently induces K_IR_2.1-Dendra2 clustering in CHO-KD cells.
