# Supplementary material for: Chronic Propafenone Application Increases Functional KIR2.1 Expression In Vitro
Source: Pharmaceuticals (Basel). 2023 Mar 7;16(3):404. doi: 10.3390/ph16030404 (PMC10056987; doi:10.3390/ph16030404)
Supplement: Supplementary file 1 [file pharmaceuticals-16-00404-s001.zip › Supporting information-revised.pdf]

pharmaceuticals

Supporting information

Chronic propafenone application increases functional $K_{IR}2.1$ expression in vitro

Encan Li[1], Willy Kool[1], Liset Woolschot[1], Marcel A.G. van der Heyden [1,*]

[1] Department of Medical Physiology, Division of Heart & Lungs, University Medical Center Utrecht, Yalelaan 50, 3584 CM, Utrecht, The Netherlands
* Correspondence: m.a.g.vanderheyden@umcutrecht.nl; Tel.: +31 88 7558900

Content

1. Figure S1: Acute effect of propafenone
2. Video S1: Propafenone time-dependently induces $K_{IR}2.1$-Dendra2 clustering in CHO-KD cells

pharmaceuticals

MDPI

1.

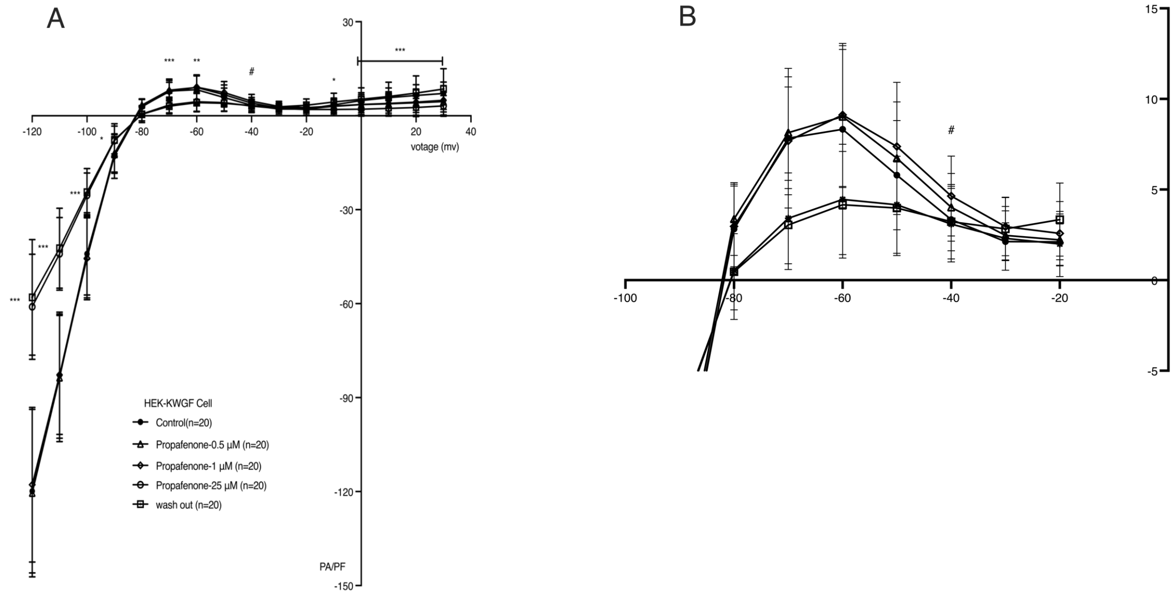

Figure S1: Acute effect of propafenone. (A) Low doses of propafenone acutely increase outward IK_1 in HEK-KWGF cells and a higher concentration of propafenone acutely blocked the IK_1. Current/voltage graph with mean values and SD of raw data of whole-cell patch-clamp experiments in HEK-KWGF cells. (B) Figure B is an enlarged part of Figure A between -90 mV to -20 mV. Each treatment condition (Propafenone 0.5 μM, 1 μM, 25 μM, Washout) lasted for 5 minutes, n=20. $*P < 0.05$, $**P < 0.01$, $***P < 0.001$ propafenone-25 μM *vs.* Control. # $P < 0.05$ propafenone-1 μM *vs.* Control.

2. Video S1: Propafenone time-dependently induces K_{IR}2.1-Dendra2 clustering in CHO-KD cells. Cloned CHO-KD cells were incubated for 24 h with DMSO or different periods (3, 6, 24, or 48h) with 25 μM propafenone. Video S1-1(CHO-KD cells treated with DMSO) shows that K_{IR}2.1-Dendra2 is present in the plasma membrane and the cell's interior as many little clusters. These clusters moved fast in all directions. Video S1-2 shows that K_{IR}2.1-Dendra2 moved slower after being treated with propafenone for 3 h and big clusters appeared in the cells' interiors. Video S1-3 to Video S1-5 show that more and larger clusters became visible in the cells and the larger clusters of K_{IR}2.1-Dendra2 displayed less movement, while the remaining small clusters moved fast. In Video-5, the cells were treated for 48h with propafenone, and multivesicular bodies (MVBs) became present in the clusters, indicating that K_{IR}2.1-Dendra2 accumulated in late endosomes.

The video materials were in file Video S1.
